# Supplementary material for: Complexome profiling of the Chlamydomonas psb28 mutant reveals TEF5 as an early PSII assembly factor
Source: Plant Cell. 2025 Mar 17;37(6):koaf055. doi: 10.1093/plcell/koaf055 (PMC12164590; doi:10.1093/plcell/koaf055)
Supplement: koaf055_Supplementary_Data [file koaf055_supplementary_data.zip › psb28_interactive.zip!/plots/intensity/qualityPlots/peptides/_Cre02.g099800.t1.1.html]

### Cre02.g099800.t1.1

Trivial name:   
SeqCoverage [median]: 19.90  
Mass: 34.12 kDa  
  
 

| Sequence | MissedCleavage | Mass [Da] | wtIntensity | muIntensity | Score |
| --- | --- | --- | --- | --- | --- |
| GADLAAGPGLQFR | 0 | 1271.66 | 811.55 | 0.00 | 89.25 |
| IVESNTAAVPDLDTLAR | 0 | 1783.93 | 528.43 | 1241.88 | 62.04 |
| LMASQGMPVGLSLGAAGAAAAGAGGR | 0 | 2241.14 | 776.49 | 0.00 | 72.03 |
| RIVESNTAAVPDLDTLAR | 1 | 1940.03 | 1521.62 | 2371.15 | 65.27 |
| SLEAMSADIK | 0 | 1063.52 | 0.00 | 607.06 | 69.42 |
|  |  |  | 3638.1 | 4220.1 |  |
